# Founding Editorial: Embryology — An Integrated Approach

**DOI:** 10.1100/tsw.2001.298

**Published:** 2001-10-26

**Authors:** Michael Richardson, Roger Keynes, Paula Mabee, Lynne Selwood

**Affiliations:** 1Institute of Evolutionary and Ecological Sciences, Leiden University, Kaiserstraat 63, Postbox 9516, 2300 RA Leiden, The Netherlands; 2University of Cambridge, Department of Anatomy, Downing Street, Cambridge, CB2 3DY, UK; Department of Biology, University of South Dakota, Vermillion, SD 57069, USA; Department of Zoology, University of Melbourne, Victoria 3010, Australia

**Keywords:** comparative study, teratology, embryology, developmental biology, evolution

## Abstract

We introduce the Embryology domain of TheScientificWorld and outline the scope and aims. We argue for an interdisciplinary approach to problems in develop-mental biology. Three areas are identified as being of particular relevance to this domain: evolutionary developmental biology, teratology, and descriptive or experimental embryology.

### BACKGROUND

Embryology aims to study the unfolding of a life, as the fertilised egg is transformed into an adult. This process, called development or ontogeny, is of great intrinsic interest. It is also central to our understanding of how animal body plans evolve; many differences between species reflect the action of natural selection on developmental mechanisms. In biomedical research, embryology provides explanations for congenital malformations and their causative factors (including teratogens).

At first sight, evolutionary developmental biology and teratology are distant cousins. The first is a branch of zoology, and helps satisfy our curiosity about the origins of life. The second is a discipline allied to clinical medicine and healthcare. Nonetheless, there are some links between the two fields. Both use a comparative approach to some extent, the comparisons being made between species, or between normal and abnormal development; both aim to understand the generation of form (morphogenesis); and both require detailed understanding of normal developmental processes in different species.

The discipline of embryology is often called 'developmental biology'. There is no real distinction between these terms, although 'embryology' is most often applied to the study of development at higher levels of organisation. Developmental biology, by contrast, is the preferred term for more molecular and cellular studies. To some extent this distinction is unfortunate; there is growing recognition that biological problems need to be understood at all levels of organisation, using integrative or multidisciplinary approaches. An example of this is the successes with which molecular approaches have been applied to problems in classical embryology and morphology.

*TheScientificWorld* is a newly established venture, and we believe that its growth will be driven in large part by the quality of the manuscripts that it publishes. High quality articles will attract the interest and the confidence of scientists around the world. In *Embryology*, as in the other domains, quality will be carefully controlled through the peer review process.

### SCOPE AND EMPHASIS

The interdisciplinary spirit of *TheScientificWorld* discourages rigid boundaries between domains. Intending authors can chose the most appropriate domain for their manuscript according to the structure of the editorial panel. We will therefore consider any manuscript which provides insight into mechanisms of normal or abnormal development in animals. Although our coverage is broad, we do have areas of special interest.

#### Evolutionary Developmental Biology

Studies at any level of organisation, from the genetic to population levels, will be considered. Evolution and development ('evo devo') has been an up-and-coming new subject for some years now, and people are wondering where the future lies. We believe that new directions include the application of phylogenetic methodology to developmental studies, genomics, and proteomics.

#### Teratology

This subject area encompasses congenital malformations and the teratogens which can cause them. We will consider studies of the mechanisms underlying the development of malformations with relation to normal development in any animal species. Descriptive studies of particular malformations or syndromes will also be considered. Routine descriptions of the effects of single agents, which do not offer embryological insights, will not be given high priority.

#### Morphological and Experimental Developmental Biology

Studies in this category have potential to inform our understanding of normal and abnormal development, and the evolution of developmental mechanisms. They also provide the essential foundations for studies of abnormal development: congenital malformations can only be understood if normal development is understood.

We expect an increasing number of developmental biologists to be attracted by what *TheScientificWorld* offers, including rapid publication, high standards of peer review, the retention of copyright by the author, and free colour and multimedia content. We are continuing to recruit the Editorial Board, but we extend a warm welcome to the scientists who have already shown their commitment and support for this valuable publishing venue.

## *EMBRYOLOGY DOMAIN*

### PRINCIPAL EDITOR

**Prof. Michael Richardson**
Institute of Evolutionary and Ecological Sciences, Leiden University, Kaiserstraat 63, Postbox 9516, 2300 RA Leiden, The Netherlands
Email: richardson@rulsfb.leidenuniv.nl

### ASSOCIATE EDITORS

**Prof. Roger Keynes,** University of Cambridge, Department of Anatomy, Downing Street, Cambridge, CB2 3DY, U.K.
**Prof. Paula Mabee,** Department of Biology, University of South Dakota, Vermillion, SD 57069, U.S.
**Prof. Lynne Selwood,** Department of Zoology University of Melbourne, Victoria 3010, Australia

### EDITORIAL BOARD MEMBERS

**Prof. Robert H. Anderson,** University College London, U.K.
**Prof. Nigel Brown,** St. George's Hospital Medical School, London, U.K.
**Dr. Ann Burke,** Wesleyan University, U.S.
**Dr. Martin Cohn**, University of Reading, U.K
**Dr. Tony Durston,** Hubrech Laboratorium, The Netherlands
**Dr. Frietson Galis**, University of Leiden, The Netherlands
**Prof. Wout Lamers**, University of Amsterdam, The Netherlands
**Prof. Alessandro Minelli**, University of Padova, Italy
**Prof. Dr. Lennart Olsson**, Fredrich-Schiller Universität Jena, Germany
**Prof. Gerhard Scholtz,** Humboldt University of Berlin, Germany
**Prof. Glenda Wright**, University of Prince Edward Island, Canada
